# The Role of Leadership in a Digitalized World: A Review

**DOI:** 10.3389/fpsyg.2019.01938

**Published:** 2019-08-27

**Authors:** Laura Cortellazzo, Elena Bruni, Rita Zampieri

**Affiliations:** ^1^Department of Management, Ca' Foscari University, Venice, Italy; ^2^Department of Business and Management, LUISS Guido Carli University, Rome, Italy

**Keywords:** leadership, e-leadership, digital transformation, digital technology, literature review, skills, ethics, virtual teams

## Abstract

Digital technology has changed organizations in an irreversible way. Like the movable type printing accelerated the evolution of our history, digitalization is shaping organizations, work environment and processes, creating new challenges leaders have to face. Social science scholars have been trying to understand this multifaceted phenomenon, however, findings have accumulated in a fragmented and dispersed fashion across different disciplines, and do not seem to converge within a clear picture. To overcome this shortcoming in the literature and foster clarity and alignment in the academic debate, this paper provides a comprehensive analysis of the contribution of studies on leadership and digitalization, identifying patterns of thought and findings across various social science disciplines, such as management and psychology. It clarifies key definitions and ideas, highlighting the main theories and findings drawn by scholars. Further, it identifies categories that group papers according to the macro level of analysis (e-leadership and organization, digital tools, ethical issues, and social movements), and micro level of analysis (the role of C-level managers, leader's skills in the digital age, practices for leading virtual teams). Main findings show leaders are key actors in the development of a digital culture: they need to create relationships with multiple and scattered stakeholders, and focus on enabling collaborative processes in complex settings, while attending to pressing ethical concerns. With this research, we contribute to advance theoretically the debate about digital transformation and leadership, offering an extensive and systematic review, and identifying key future research opportunities to advance knowledge in this field.

## Introduction

The findings of the latest Eurobarometer survey show the majority of respondents think digitalization has a positive impact on the economy (75 percent), quality of life (67 percent), and society (64 percent) (European Commission, 2017). Indeed, people's daily lives and businesses have been highly transformed by digital technologies in the last years. Digitalization allowed to connect more than 8 billion devices worldwide (World Economic Forum, 2018), modified information value and management, and started to change the nature of organizations, their boundaries, work processes, and relationships (Davenport and Harris, 2007; Lorenz et al., 2015; Vidgen et al., 2017).

Digital transformation refers to the adoption of a portfolio of technologies that, at varying degrees, have been employed by the majority of firms: Internet (IoT), digital platforms, social media, Artificial Intelligence (AI), Machine Learning (ML), and Big Data (Harvard Business Review Analytic Services, 2017). These tools and instruments are “rapidly becoming as infrastructural as electricity” (Cascio and Montealegre, 2016, p. 350). At macro levels, the shift toward different technologies is setting the agenda for new mechanisms of competition, industry structures, work systems, and relations to emerge. At the micro level, the digitalization has impacted on business dynamics, processes, routines, and skills (Cascio and Montealegre, 2016).

Across different sectors and regardless of organization size, companies are converting their workplaces into *digital* workplaces. As observed by Haddud and McAllen (2018), many jobs now involve extensive use of technology, and require the ability to exploit it at a fast pace. Yet, digitalization is being perceived both as a global job destroyer and creator, driving a profound transformation of job requirements. In result, leaders need to invest in upskilling employees, in an effort to support and motivate them in the face of steep learning curves and highly cognitively demanding challenges. Moreover, increased connectivity and information sharing is contributing to breaking hierarchies, functions and organizational boundaries, ultimately leading to the morphing of task-based into more project-based activities, wherein employees are required to directly participate in the creation of new added value. As such, the leadership role has become vital to capture the real value of digitalization, notably by managing and retaining talent via better reaching for, connecting and engaging with employees (Harvard Business Review Analytic Services, 2017; World Economic Forum, 2018). However, leaders need to be held accountable for addressing new ethical concerns arising from the dark side of digital transformation. For instance, regarding the exploitation of digitalization processes to inflict information overload onto employees, or to further blur the lines between one's work and personal life.

In the last few decades, leadership scholars have been trying to monitor the effects of digitalization processes. Part of the academic debate has been focused on the role of leaders' ability to integrate the digital transformation into their companies and, at the same time, inspire employees to embrace the change, which is often perceived as a threat to the current status quo (Gardner et al., 2010; Kirkland, 2014). To bring clarity to this debate, the construct of e-leader has been introduced to describe a new profile of leaders who constantly interact with technology (Avolio et al., 2000; see also Avolio et al., 2014 for a review). Accordingly, e-leadership is defined as a “social influence process mediated by Advanced Information Technology (AIT) to produce a change in attitudes, feelings, thinking, behavior, and/or performance with individuals, groups, and/or organizations” (Avolio et al., 2000, p. 617).

Despite the increasing interest in discussing the relationship between digital technology and leadership, contributions have accumulated in a fragmented fashion across various disciplines. This fragmentation has made scholars struggle “to detect larger patterns of change resulting from the digital transformation” (Schwarzmüller et al., 2018, p. 114). It also suggests that scholars have relied on multiple theoretical models to explain the phenomenon. Indeed, if, on one hand, it is clear that organizations are changing due to technological improvements, on the other hand, the way in which the transformation is occurring remains under debate. Furthermore, due to the fast-changing development and implementation of digital technology, there is a need to continuously update and consider the latest contributions to the topic.

This article addresses the aforementioned issues by offering a systematization of the literature on digitalization and leadership that has been accumulating across different disciplines, while adopting an interdisciplinary approach and providing a systematization of articles from different fields that analyze digitalization and leadership. Specifically, the present article reviews the literature on how the advent of digital technologies has changed leaders and leadership roles. Moreover, it structures and summarizes the literature, considering both theoretical frameworks and empirical findings, and fostering the understanding of both the content of the debate and its practical underpinnings. Lastly, reflecting on the findings of this review, we offer suggestions for future directions of research.

The present review draws on the following boundary conditions. First, we relied on a broad definition of leadership, in which the leader is understood as a person who guides a group of people, an organization, or empowers their transformational processes. Second, we excluded studies referring to market or industry leaders, in which the leader is represented by an organization. Third, we considered studies that clearly referred to a digital or technological transformation. Fourth, we did not include studies in which there was not a clear link between information technology and leadership (e.g., city leaders protecting the physical and digital infrastructures of urban economies regarding climate change). Therefore, our review was guided by the following research questions: *(i) What are the main theoretical frameworks guiding the academic discussion on digital transformation and leadership? (ii) What are the main categories emerging from the contributions that address the relationship between digital transformation and leadership? And (iii) Which are the main future directions of research that scholars should consider?*

This paper is structured as follows: First, it describes the methodology used; Second, it proposes a classification of findings based on theoretical frameworks and content. Finally, it describes implications of our findings for both research and practice, and proposes directions for future research.

## Research Design

The aim of this paper is to investigate how the debate on digital transformation and leadership has evolved in recent years, to identify key theories and findings, and to propose potential future directions of research. To answer our research questions, we use a mixed method approach, that involves both quantitative research through standard databases and qualitative coding (Crossan and Apaydin, 2010; Peteraf et al., 2013; Zupic and Čater, 2015).

### Data Collection

We collected papers from the Scopus database, one of the most widely used sources of scientific literature (Zupic and Čater, 2015). We also checked Web of Science and Ebsco databases in order to avoid missing articles. Because we did not find any relevant distinction between these databases regarding this topic, we chose to use Scopus only. We firstly accessed the database on September 1st, 2018.

**Figure 1:**
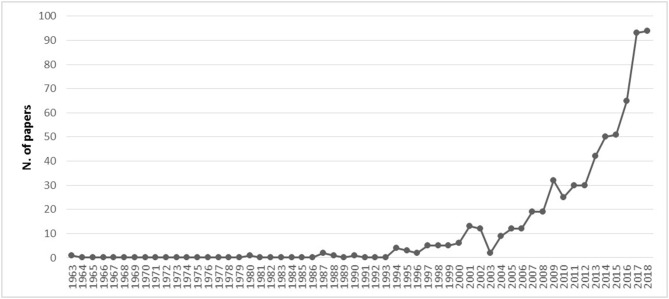
Growth of articles on leadership and digitalization.

Since our research questions concerned the academic discussion on digital transformation and leadership, the scope of our search was limited to academic articles (not only from peer-reviewed journals but also from unpublished sources, such as unpublished manuscripts). Non-academic books and other publications were outside the scope of our study and were therefore excluded from our search. Our initial search was undertaken using the basic keywords: leader^*^ AND digital^*^ OR e-leader^*^. The keywords were used as a selection criterion for the topic (title, keywords, or abstract). We searched peer-reviewed papers published in English, in journals focusing on the following subject areas: Business, Management, and Accounting; Psychology; and Social Science, without any additional selection restrictions. We decided to scan articles published in other areas than Business and Management since the topic is covered by several disciplines. These criteria resulted in an initial sample of 790 articles. The following figure (Figure 1) shows how the debate grew since 2000, and significantly expanded since 2015.

In order to avoid a potential publication bias (O'Boyle et al., 2017), and to scan recent studies that might not have had the time to go through the entire publication process, we performed a search within conference proceedings since 2015, using the same aforementioned criteria. The initial sample comprised 113 articles.

**Figure 2:**
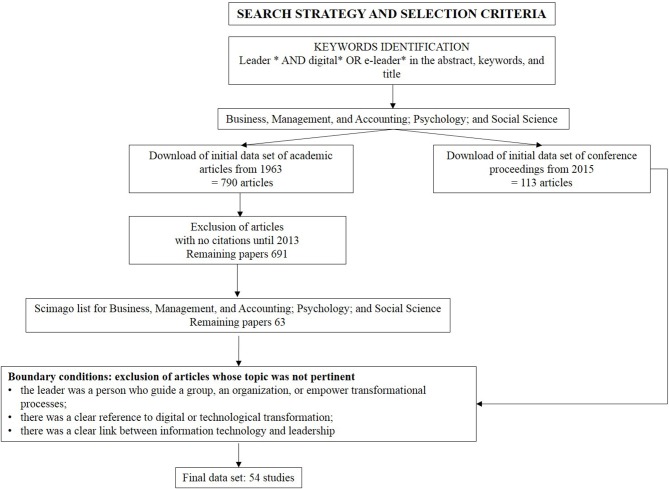
Search strategy and selection criteria.

The second step within our data collection process involved a qualitative selection of articles. We first considered publications with at least one citation among those published before 2013, seen that the number of citations is a common criterion of scientific rigor and impact in academia (Garfield, 1979, 2004; Peteraf et al., 2013). As citation-based methods may discriminate against recent publications (Crossan and Apaydin, 2010), we kept all papers published after 2013. Based on the assumption that top journals publish high quality papers, we discarded studies that were not included within the first 200 journals appearing in the Scimago list within the Management and Business, Social Science, and Psychology areas. Then, both peer-reviewed articles and conference proceedings were filtered based on the assessment of whether the abstracts were in alignment with the topic and the boundary conditions. Articles were selected based on the following criteria: (i) the leader was a person who guides a group, organization, or empowers their transformational processes; (ii) there was a clear reference to digital or technological transformation; (iii) there was a clear link between information technology and leadership. Articles that focused on either digital transformation or leadership only were excluded, as well as papers that were outside our boundary conditions, such as studies on industry leaders using digital platforms. Figure 2 summarizes the selection criteria and the boundary conditions used to scan the articles. The search criteria resulted in a final dataset of 54 studies.

### Data Analysis and Qualitative Coding

To attain a “systematic, transparent and reproducible review process” (Zupic and Čater, 2015, p. 429), and identify research streams and seminal works, we first performed a bibliometric analysis of the initial dataset of 790 articles. In order to map the origin and evolution of the academic debate on digital transformation and leadership, a systematic coding analysis was conducted on the entire set of articles. Then, the iterative reading and discussion of the final dataset of articles highlighted the following emerging categories that guided our analysis (Strauss and Corbin, 1998): (i) theoretical or empirical papers; (ii) research methodology; (iii) level of analysis (micro and macro); (iv) definition of leadership and digitalization; (v) main themes or objectives of the article; (v) main underlying theories; (vi) field of study (e.g., Management and Planning, Economics and Business, Psychology and so forth). Based on this coding scheme, the three authors independently read and coded all articles. Subsequently, they discussed their coding attribution until an agreement on the final coding of each article was reached.

## Findings

### Dataset Description

The final database comprises 54 articles, of which 42 are peer-reviewed papers published by 33 journals, while the remaining 12 papers are conference proceedings (see Table 1).

**Table 1:** Dataset citations, source, level of analysis and empirical/theoretical approach.

**Paper**	**Number of citations**	**Source**	**Level of analysis**	**Empirical or theoretical approach**
Avolio et al., 2000	222	Leadership Quarterly	Macro	Theoretical
McAfee and Brynjolfsson, 2008	174	Harvard Business Review	Macro	Empirical
Rosenbloom, 2000	167	Strategic Management Journal	Micro	Empirical
Hambley et al., 2007	112	Organizational Behavior and Human Decision Processes	Micro	Empirical
Avolio et al., 2014	45	Leadership Quarterly	Macro	Theoretical
Robin et al., 2011	42	Academic Medicine	Macro	Theoretical
Lee and Man Chan, 2016	27	Information Communication and Society	Micro	Empirical
Horner-Long and Schoenberg, 2002	26	European Management Journal	Macro	Empirical
Agarwal et al., 2014	24	Information Communication and Society	Macro	Empirical
Lee, 2009	19	International Journal of Project Management	Macro	Theoretical
Kodama, 2007	18	Technovation	Macro	Empirical
Morgareidge et al., 2014	15	Frontiers of Architectural Research	Macro	Empirical
Heinz et al., 2006	14	Team Performance Management: An International Journal	Macro	Empirical
Diamante and London, 2002	13	Journal of Management Development	Micro	Theoretical
Lynn Pulley and Sessa, 2001	12	Industrial and Commercial Training	Micro	Empirical
Lu et al., 2014	12	Computers in Human Behavior	Micro	Empirical
Gordon, 2007	11	Information Communication and Society	Macro	Theoretical
Geoffrion, 2002	8	Production and Operations Management	Macro	Theoretical
Gerbaudo, 2017	7	Information Communication and Society	Macro	Empirical
Gerth and Peppard, 2016	6	Business Horizons	Micro	Empirical
Weiner et al., 2015	6	Journal of Healthcare Management	Macro	Empirical
Tsai and Men, 2017	5	New Media and Society	Micro	Empirical
Sullivan et al., 2015	5	Network Science	Micro	Empirical
Berman and Korsten, 2014	4	Strategy and Leadership	Macro	Empirical
Jawadi et al., 2013	4	Human Systems Management	Micro	Empirical
Obschonka et al., 2017	3	Journal of Business Venturing Insights	Micro	Empirical
Bartol and Liu, 2002	3	Research in Personnel and Human Resources Management	Macro	Theoretical
Toepfl, 2018	3	Information Communication and Society	Macro	Empirical
Henttonen et al., 2012	3	Journal of Technology Management and Innovation	Micro	Empirical
Coutu, 2000	3	Harvard Business Review	Micro	Empirical
Penney, 2017	2	Journal of Communication	Micro	Empirical
Eyal, 2016	2	Political Communication	Macro	Empirical
Sousa and Rocha, 2018	1	Journal of Business Research	Micro	Empirical
Grafström and Falkman, 2017	1	Journal of Organizational Change Management	Micro	Empirical
Bolden and O'Regan, 2016	1	Journal of Management Inquiry	Micro	Theoretical
Roman et al., 2018	0	Public Administration Review	Micro	Empirical
Richardson and Sterrett, 2018	0	Educational Administration Quarterly	Micro	Empirical
Gupta and Pathak, 2018	0	Journal of Organizational Change Management	Micro	Empirical
Schwarzmüller et al., 2018	0	Management Revue	Micro	Empirical
David and Baden, 2018	0	Information Communication and Society	Macro	Empirical
Bakardjieva et al., 2018	0	Information Communication and Society	Macro	Empirical
Boe and Torgersen, 2018	0	Frontiers in Psychology	Macro	Empirical
Dimitrov, 2018	1	Conference Paper—Symposium—University of Phoenix: Envisioning Future Leadership: Utopia, Dystopia, or More of the Same?	Micro	Theoretical
Petrucci and Rivera, 2018	0	Conference Paper—Symposium—University of Phoenix: Envisioning Future Leadership: Utopia, Dystopia, or More of the Same?	Micro	Theoretical
Stolze et al., 2018	0	Conference Paper—European Conference on Innovation and Entrepreneurship	Macro	Empirical
Haddud and McAllen, 2018	0	Conference Paper—Portland International Conference on Management of Engineering and Technology	Macro	Theoretical
Foerster and Duchek, 2018	0	Conference Paper—Annual Meeting of the Academy of Management	Micro	Theoretical
Wang et al., 2018	0	Conference Paper—International Conference on Electronic Business	Micro	Empirical
Jones, 2017	0	Conference Paper—International Conference on Management Leadership and Governance	Micro	Empirical
Bekkhus and Hallikainen, 2017	0	Conference Paper—Conferencia da Associacao Portuguesa de Sistemas de Informacao	Micro	Theoretical
Prince, 2017	0	Conference Paper—International Conference on Electronic Business	Micro	Theoretical
Van Outvorst et al., 2017	0	Conference Paper—International Conference on Management Leadership and Governance	Micro	Theoretical
Bygstad et al., 2017	0	Conference Paper—Scandinavian Conference on Information Systems	Macro	Empirical
Gheni et al., 2016	0	Conference Paper—IEEE Conference on e-Learning, e-Management and e-Services	Micro	Theoretical

Regarding the peer reviewed articles in our dataset, most of them stem from Economic, Business and Management (22 articles), and Information and Communication Sciences (10 articles). Only three studies come from the Psychology discipline. As for the sources wherein these articles are published, we count two journals that specifically address the leadership field, such as “The Leadership Quarterly” and “Strategy and Leadership”, whereas the remaining 31 other journals are spread across areas such as Economics, Business and Management, Information and Communication Sciences, Psychology, Educational, Heath and Political Sciences. The novelty of the topic and the breadth of journals in which it is published confirms that the field of digital transformation and leadership has garnered interest from several difference disciplines. Such fragmentation of the literature and the different perspectives it has enabled, justifies the need for systematization and alignment of future research.

As for the conference proceedings, half of the articles come from international and peer-reviewed conferences advancing the debate of digital transformation in business, such as the International Conference on Electronic Business, the Scandinavian Conference on Information Systems, the IEEE Conference on e-Learning, e-Management and e-Services.

Among the top five most cited articles in our sample, three come from journals that specifically relate to Human Resources: “Leadership Quarterly” and “Organizational Behavior and Human Decision Processes.” In these articles the authors focus on the characteristics of digital leaders in terms of roles and behaviors, stressing the idea that technology is deeply changing the way in which leaders conceive communication and cope with their followers (Avolio et al., 2000, 2014; Horner-Long and Schoenberg, 2002; Hambley et al., 2007).

As shown in Figure 1, the early 2000s witnessed an initial interest in the topic, when pioneering work began to consider the changes that digitalization brings in the area of leadership and how the concept and practice of leadership are affected by new technologies (Avolio et al., 2000; Coutu, 2000). However, it has been mostly over the last decade that the topic garnered seesawing attention. It is plausible to assert that the much stronger impact that technological development has had within organizations recently, and the expectation that technological evolution will be even more disruptive in the near future, has accelerated the interest on the topic. Indeed, while all peer-reviewed articles in our sample are from 2000 on, 60 percent were published after 2014. As for conference proceedings, we only considered the contributions presented after 2015 in order to understand how the debate has been developing in recent years.

Regarding the level of analysis (micro vs. macro), the majority of contributions within our sample are at the micro-level (30 articles), while 24 adopt a macro perspective. Within the latter, it is interesting to notice that a considerable number of articles do not pertain to the management field. As to the type of contribution, the majority of articles in our sample (37) are empirical studies, while only a few articles are conceptual. This imbalance reveals there is still a lack of theorization about the impact of technology on leadership. Nevertheless, in the next session we systematize the main theoretical frameworks that have been used to address this topic.

### Main Theoretical Frameworks

The analysis of the theoretical content of our dataset highlighted that only a small set of studies explicitly refers to the extant theoretical frameworks describing the impact of digital transformation on leadership. Advanced information technologies theory (Huber, 1990), according to which the adoption of information technologies influences changes in organization structure, information use, and decision-making processes, is used as common ground. Scholars agree on the high impact of technology in leadership behavior and identify Information Technologies (IT) developments as a driver for creating disruptive changes in businesses and in leadership roles across different organizational functions (Bartol and Liu, 2002; Geoffrion, 2002; Weiner et al., 2015; Sousa and Rocha, 2018). These changes are so dramatic that scholars started to adopt a new terminology to characterize the e-world, e-business and e-organizations (Horner-Long and Schoenberg, 2002). Recent studies have been discussing the notion of digital ubiquity (Gerth and Peppard, 2016; Schwarzmüller et al., 2018), describing the pervasive proliferation of technology (Roman et al., 2018). With this term, scholars refer to a context in which technological equipment is prevalent and constantly interacts with humans. It describes a scenario in which “computer sensors (such as radio frequency identification tags, wearable technology, smart watches) and other equipment (tablets, mobile devices) are unified with various objects, people, information, and computers as well as the physical environment” (Cascio and Montealegre, 2016, p. 350).

In terms of leadership theoretical frameworks, scholars seem to turn to a plethora of different theories and definitions. Horner-Long and Schoenberg (2002) contrapose two main theoretical approaches: universal theories and contingency theories. The former supports the view that leaders differ from other individuals due to a generic set of leadership traits and behaviors which can be applied to all organizations and business environments (see for example Lord et al., 1986; Kirkpatrick and Locke, 1991). The latter argues that, in order to be effective, leadership should adopt a style and behaviors that match the context (e.g., Tannenbaum and Schmidt, 1973; Goleman, 2000). The authors empirically explore leadership profile characteristics, comparing e-business leaders and leaders from traditional bricks and mortar organizations. Results do not clearly support any of the two approaches. They suggest that in both contexts most of leadership characteristics are equally valued. However, certain characteristics distinguish e-world leaders from leaders in traditional industries. While Horner-Long and Schoenberg (2002) analyze leader profile differences across industries, Richardson and Sterrett (2018) adopt a longitudinal design, exploring how digital innovations influenced the role of technology-savvy K-12 district leaders across time. They base their work on a unified model of effective leadership practices that influence learning (Hitt and Tucker, 2016). Although the leadership practice model is maintained across time, the authors recognize some shifts in the way those practices are implemented.

Only Obschonka et al. (2017) specifically adopt a universal perspective, drawing from trait approach theory (Stogdill, 1974). By analyzing the language used to communicate via Twitter, the authors identify the personality characteristics that distinguish the most successful managers and entrepreneurs.

Heinz et al. (2006) follow a contingency approach, emphasizing the need to take into account the context and consider situational aspects that can influence leadership and cooperation practices.

Most studies in our sample assume that the change in context due to technological advancement may influence leadership. According to Lu et al. (2014, p. 55), it cannot be assumed that “leadership skills identified in offline context should be transferred to virtual leadership without any adjustment.”

However, some authors make this assumption tacitly (e.g., Schwarzmüller et al., 2018), without explicitly addressing any related theoretical framework. Bolden and O'Regan (2016, p. 439) report that “there is no one approach to leadership,” since leadership is context specific and must to be adapted to the needs of the day. Similarly, Lu et al. (2014) maintain that effective leadership behaviors are determined by the situation in which leadership is developed.

To address the diversity of situations and contexts, Jawadi et al. (2013) overcome the limits of a pure contingency approach and embrace complexity, adopting the framework of leadership behavioral complexity theory (Denison et al., 1995). In a context characterized by complex and unanticipated demands, a leader needs to develop a behavioral repertoire that allows dealing with contradictory and paradoxical situations (Denison et al., 1995). As contingencies are evolving so rapidly as to be considered in a state of flux, an effective leader needs to be able to conceive and perform multiple behaviors and roles.

Avolio et al. (2000, 2014), make a step forward in defining the role of context.

Similarly to Bartol and Liu (2002), the authors adopt a structurational perspective (Adaptive Structure Theory) (AST; DeSanctis and Poole, 1994) as the main theoretical framework. According to their point of view, digital technologies and leadership reciprocally influence and change each other in a recursive relationship. In their perspective, not only technology influences leadership, but also leaders appropriate technology, and it is through the interaction between information technology and organizational structures that the effect of technology on individuals, groups, and organizations emerges. In this view, the context is not only shaping and shaped by leaders; it is part and parcel of the construct of e-leadership itself. Avolio et al. (2000, 2014) remarkably paved the way for the conceptualization of e-leadership, which has since been adopted by many other authors to inform their studies (Avolio et al., 2000; Lynn Pulley and Sessa, 2001; Roman et al., 2018).

Similarly, Orlikowski (1992) develop a Structurational Model of Technology, whereby technology influences the context in which actors perform but is also designed and socially constructed by its users (Van Outvorst et al., 2017).

Looking at leaders' relationships with their teams, scholars refer to the following main theories: transactional leadership theory, transformational leadership theory (Burns, 1978; Bass, 1981, 1985), and leader-member exchange theory (LMX; Graen and Scandura, 1987). Transactional and transformational leadership are among the most influential and discussed behavioral leadership theories of the last decade (Diaz-Saenz, 2011). They distinguish transformational leaders, who focus on motivating and inspiring followers to perform above expectations, from transactional leaders, who perceive the relationship with followers as an exchange process, in which follower compliance is gained through contingent reinforcement and rewards (Bass, 1985). Previous studies reveal that leadership styles may influence virtual team interactions and performance (e.g., Sosik et al., 1997; Sosik et al., 1998; Kahai and Avolio, 2006). As such, Hambley et al. (2007) explore the effects of transactional and transformational leadership on team interactions and outcomes, comparing teams interactions across different communication media: face-to-face, desktop videoconference, or text-based chat. Likewise, Lu et al. (2014) compare virtual and offline interactions, drawing on transactional and transformational leadership theories to understand whether leadership styles of individuals playing in Massive Multiplayer Online Games (MMOGs) can be associated to their leadership status in offline contexts. However, this association is found to be significant only with offline leadership roles in voluntary organizations, not in companies. Results in Hambley et al. (2007) also show that the association between leadership style and team interaction and performance does not depend on the communication medium being used.

While transactional and transformational leadership theories adopt a behavioral perspective in which the focal point is the leader behavior with regards to the follower, leader-member exchange theory (LMX) introduces a dyadic point of view. Leader-member exchange theory focuses on the nature and quality of the relationship between leaders and their team members. The quality of this relationship, which is characterized by trust, respect, and mutual obligation, is thought to predict individual, group and organizational outcomes (Gerstner and Day, 1997). Jawadi et al. (2013) use the concept of leader-member exchange as a dependent variable, exploring how multiple leadership roles influence cooperative and collaborative relationships in virtual teams. Bartol and Liu (2002) build on leader-member exchange theory to suggest policies and practices HRM professionals can use to implement IT-information sharing and positively influence employee perceptions.

The democratization of informational power gave momentum to distributed power dynamics. Moving beyond the centrality of the sole vertical leader, the shared leadership approach emphasizes the role of teams as potential source of leadership (Pearce, 2004; Ensley et al., 2006; Pearce et al., 2009). Shared leadership is “a manifestation of fully developed empowerment in teams” (Pearce, 2004, p. 48) in which leadership behaviors that “guide, structure, or facilitate the group may be performed by more than one individual, and different individuals may perform the same leadership behaviors at different times” (Carte et al., 2006: p. 325).

Acknowledging the relevance of increased connectivity in the digital era, some studies underscore the importance to take into account a network perspective. Lynn Pulley and Sessa (2001) contrapose the industrial economy to the current networked economy. Bartol and Liu (2002) define networked organizations as those organizations characterized by three major types of connectivity: inter-organizational (also known as boundaryless; Nohria and Berkley, 1994), intra-organizational, and extra-organizational. Kodama (2007) views the organization as the integration of different types of networked strategic communities, wherein knowledge is shared and assessed. Sullivan et al. (2015) use a network representation to depict shared leadership. Gordon (2007) explores how the network is embedded in the concept of web that is currently accepted.

### The Macro Perspective of Analysis: Main Categories

The studies on digitalization and leadership that adopt a macro-perspective of analysis can be classified in four different categories, according to whether they focus on: (1) The relationship between e-leaders and organizations; (2) How leaders adopt technology to solve complex organizational problems; (3) The impact of digital technologies on ethical leadership; or (4) The leader's use of digital technologies to influence social movements.

#### The Relationship Between E-Leaders and Organizations

The studies within our sample that take a macro or organizational-level approach are considerably less than those which investigate the micro dynamics occurring within organizations. A summary is shown in Table 2. This imbalance is probably due to the relatively greater urgency and challenge to understand the role of leaders and leadership in guiding and implementing the digitalization process within organizations, rather than what new forms of organizations are emerging as a result of the digital transformation. As observed by a recent Harvard Business Review Analytic Services report (2017), leaders have increasingly become the key players in driving positive results from the investments on digital tools and technologies.

**Table 2:** Main categories summary.

**Category**	**Authors**
**MACRO PERSPECTIVE**	
E-leaders and organizations	Avolio et al., 2000; Lynn Pulley and Sessa, 2001; Kodama, 2007; Henttonen et al., 2012; Berman and Korsten, 2014; Van Outvorst et al., 2017
Digital tools and organizations	Bartol and Liu, 2002; Morgareidge et al., 2014; Weiner et al., 2015; Prince, 2017; Haddud and McAllen, 2018
Organizations and ethics	Lee, 2009; Berman and Korsten, 2014; Jones, 2017
Leadership and digital tools: insights from social movement studies	Agarwal et al., 2014; Lee and Man Chan, 2016; Gerbaudo, 2017; Bakardjieva et al., 2018; David and Baden, 2018; Toepfl, 2018
**MICRO PERSPECTIVE**	
The evolution of C-level roles	Gerth and Peppard, 2016; Bekkhus and Hallikainen, 2017; Grafström and Falkman, 2017; Obschonka et al., 2017; Tsai and Men, 2017
Leaders' skills in the Digital Era	Coutu, 2000; Rosenbloom, 2000; Lynn Pulley and Sessa, 2001; Diamante and London, 2002; Horner-Long and Schoenberg, 2002; Robin et al., 2011; Avolio et al., 2014; Lu et al., 2014; Bygstad et al., 2017; Boe and Torgersen, 2018; Dimitrov, 2018; Foerster and Duchek, 2018; Petrucci and Rivera, 2018; Roman et al., 2018; Schwarzmüller et al., 2018; Sousa and Rocha, 2018; Stolze et al., 2018; Wang et al., 2018
Leading virtual teams	Avolio et al., 2000, 2014; Lynn Pulley and Sessa, 2001; Bartol and Liu, 2002; Hambley et al., 2007; Lee, 2009; Jawadi et al., 2013; Sullivan et al., 2015; Gheni et al., 2016; Gupta and Pathak, 2018; Roman et al., 2018; Schwarzmüller et al., 2018

In the last few years, scholars have begun to adopt the construct of e-leader in order to specifically refer to those leaders who have initiated a massive process of digitalization in their organizations. Despite the call to understand how organizations and e-leaders are intertwined, few studies provide an empirical explanation of the new organizational configurations emerging from the interaction between technology and the human/social system. Berman and Korsten (2014) is one among the few. By surveying a large sample of CEOs, running companies of different sizes and across 64 countries and 18 industries, the authors showed that outperforming organizations had leaders that created open, connected and highly collaborative organizational cultures. The authors suggest future leaders should base their organizations on three pillars: (1) Assuring a highly connected and open working environment at any hierarchical levels and units in organizations; (2) Engaging customers by gathering knowledge about the whole person; and (3) Establishing more integrated and networked relationships with partners and competitors (Berman and Korsten, 2014). They posit these three pillars transform the organizations at all levels. This implies organizations are becoming boundaryless, at both the internal and external levels. Further, the organizational structure is no longer a static feature, but an ongoing process (Van Outvorst et al., 2017). While a shift toward an ecological perspective— one where organizations' boundaries are loose and permeable—requires higher coordination, collaboration and individual responsibility, it also enhances innovative capabilities (Lynn Pulley and Sessa, 2001). According to Kodama (2007), managers at any level can foster innovation if they go beyond the formal organization, to create real or virtual networks among internal and/or external communities of practice. These communities of practice enable a more agile response to change, promoting the free-flow of information and breaking down information silos (Petrucci and Rivera, 2018), thereby empowering both managers and employees to integrate, transform and stimulate knowledge that fosters innovation. This way, information and communication technology enables the creation of shared information pools wherein diverse staff across the organization contribute to a collaborative and dynamic process of idea generation. Moreover, such co-generation of ideas and knowledge cultivates stronger relationships between disparate organizational units, further facilitating open innovation processes (Henttonen et al., 2012).

In sum, by breaking the organizational boundaries within and between internal and external stakeholders, the traditional leader-centered information and decision-making process is giving way to novel processes that democratize access to information and share decision power among all parties involved.

#### Digital Tools and Organizations: How Technology Enhances the Optimization of Complex Organizational Environments

Although most papers adopting a macro perspective reflect on the novel structures of organizations, they tend to underestimate the effect of digital transformation on organizational processes. That is, however, not the case with Weiner et al. (2015), who discuss how the effective achievement of operational goals relies on the fit between strategic planning and information technology, particularly in operationally complex organizations, such as hospitals. Their empirical study shows that digital tools could highly contribute in the planning and monitoring of internal processes, increasing the transparency and accountability across all levels of management, and engaging customers' trust. For instance, the intelligent use of data through sophisticated digital tools, allowed hospitals administrators to lead improvements in decision-making processes and service quality by enhancing the usage of traditional management tools, such as key performance indicators (KPIs), and storage of critical data, namely on infections and diseases. Notably, this study offers empirical evidence on the need to adopt digital technology to develop efficient internal organizational processes and guarantee high quality service to customers. In another empirical study conducted in a hospital, the authors confirmed that the use of digital tools helped leaders solve complex issues related to personnel and operational costs. Similarly to the previous study aforementioned, data were used to re-design the entire organization with the aim of optimizing the efficiency in the use of both facilities and processes (Morgareidge et al., 2014).

Leaders are responsible for verifying the suitability of technological tools being adopted or implemented in relation to the organizational needs and objectives. Moreover, while we acknowledge that digital technologies hold the potential for improving the efficiency of organizational processes, we contend that they need to be internalized and integrated within employees' routine tasks in order for organizations to minimize attritions from their adoption and fully capture its benefits.

#### Organizations and Ethics

Ethics in leadership roles has been an issue of concern to scholars especially since the emergence of the transformational leadership paradigm (Burns, 1978; Bass and Avolio, 1993). In general, ethical leadership is defined as “the demonstration of normatively appropriate conduct through personal actions and interpersonal relationships, and the promotion of such conduct to followers through two-way communication, reinforcement, and decision-making” (Brown et al., 2005, p. 120). With the advent of digital transformation and the massive use of data, scholars have begun to call into question the integrity of leaders. Indeed, the use of data and technologies exposes leaders to new dilemmas, which nature is intertwined with ethical concerns. For instance, the use of sensitive data is driving leaders' increased concerns about privacy protection and controlling mechanisms in the workplace (Kidwell and Sprague, 2009). Electronic surveillance (ES) is a way to collect data about employees and their behavior, so as to improve productivity and monitor behaviors in the workplace (Kidwell and Sprague, 2009). ES rules vary across countries and cultures. For instance, the US Supreme Court of Justice obliged employers to adopt ES to monitor employees in order to prevent sexual harassment (Kidwell and Sprague, 2009). Notwithstanding, Europe has been more concerned with individual privacy. Notably, in 1986, the Organization for Economic Cooperation and Development (OECD) approved a declaration on social aspects of technological change, whereby member states “were concerned that employers and unions ensure that workers' privacy be protected when technological change occurs” (Kidwell and Sprague, 2009, p. 199). Perhaps the boldest manifestation of this concern is the recently adopted EU General Data Protection Regulation (GDPR), which has just come into force the past May 25th, 2019.

In this scenario, leaders are required to set clear guidelines and practices that lie within national and international data security policies. In particular, they need to monitor the use of personal sensitive data, if not for the ethical concern *per se*, because if otherwise caught in unlawful data practices, their organizations' reputation, trustworthiness, and brand image could suffer irreparable damage (e.g., the recent scandal of Cambridge Analytica about an inappropriate use of personal data, has affected the reputation of all organizations involved) (Gheni et al., 2016; Jones, 2017). Leaders also need to set clear expectations for employees and act as role models for all members of the organization in order to clarify what ethical behavior regarding personal sensitive data looks like. This is especially true for organizations that strongly rely on virtual communications, as these tend to stimulate more aggressive and unethical behavior, due to their lack of face-to-face interactions (Gheni et al., 2016). Leaders, therefore, have a pivotal role in weeding out potential unethical behaviors from their organizations.

Finally, an emerging topic in leadership concerns the unlawful appropriation of technology from private and public organizations. Specifically, it refers to situations wherein technology is used for purposes other than those it had originally been intended (Jones, 2017). For instance, improper use of technology may result in unauthorized access to data and lead to cyber security breaches (Jones, 2017).

Despite the interdisciplinary relevance of ethics, the debate of ethical concerns within e-leadership seems to be currently confined to the literature on governance and information technology. Yet, there is room for more theoretical and empirical discussion about how ethics is affecting power relations, surveillance, safety perceptions in the workplace, and human resource processes.

#### Leadership and Digital Tools: Insights From Social Movement Studies

A complementary perspective of leadership and digitalization is provided by several recent studies that analyze social and political events, in particular grassroots movements such as the Occupy and Tea Party (Agarwal et al., 2014), the Umbrella Movement in China (Lee and Man Chan, 2016) and the political tensions in Russia (Toepfl, 2018). These contributions share the notion of leader as someone who directs collective action and creates collective identities (Morris and Staggenborg, 2004). These studies, mainly rooted in communication and political sciences, are certainly relevant to our review as they shed light on the social nature of leadership in the new digital era.

These studies focus on how social media and digital tools are disrupting traditional forms of leadership, altering the structure, norms and hierarchy of organizations, and creating new practices to manage and sustain consensus (David and Baden, 2018). New forms of leadership are for instance defined as *horizontal* and *leaderless* (Castells, 2012; Bennett and Segerberg, 2013). The *horizontality* defines movements and groups in which authority is dismissed, whereas *leaderless* points to the lack of power stratification among the participants (Sitrin, 2006; Gerbaudo, 2017).

In a similar vein, recent studies looking at the use of digital tools by participants in social movements, observe how power struggles were changed by new information and communication technologies (ICTs): “ICTs have transformed the power dynamics of social movement politics by challenging traditional forms of [social] organizations” (Agarwal et al., 2014, p. 327).

The single case study of the ultra-orthodox community illustrates for instance how authoritarian leadership can be broken down by digital tools and social media (David and Baden, 2018). When the leadership of a closed and conservative religious community is questioned in social media, that creates a new space to renegotiate the community's boundaries and modify its power dynamics: “the fluidity and temporality of digital media have advanced to become an influential, independent factor shaping community opinion” (David and Baden, 2018, p. 14). As such, the identity of a closed and inaccessible community and its leadership are challenged by both internal and external actors through the use of digital media.

The study of different digital tools is also considered a relevant subject matter to gain understanding about what tools are more efficient in organizing and mobilizing resources (Agarwal et al., 2014). Technology and digital tools are not value-neutral nor value free, because they influence how people organize, coordinate, and communicate with others (Hughes, 2004; Agarwal et al., 2014). For instance, the study on the Russian activists shows how the long-term success of the movement was a result of a centralized, formalized and stable network, wherein its leading representatives and other members were bonded together by a new digital tool (Toepfl, 2018). The use of digital instruments enabled the transformation of an organization that was initially chaotic into a more structured one, as they facilitated the discussion and coordination between the leader and its followers (Toepfl, 2018). This resulted in a more efficient and effective way to achieve consensus.

Taken together, these studies show how technology is far from being a neutral instrument. Rather, digital tools influence power dynamics in any type of organization (e.g., flat, bureaucratic or networked), and at any level. If on one hand, digital tools can lead to the de-structuring of extant hierarchies and challenge organizational boundaries and rules, on the other hand, they can be used as communication and coordination mechanisms that allow leaders to build structured networks from scratch and, through them, reinforce their power.

In sum, these studies stress that, despite the participatory dynamics that characterize social movements, power struggles and hierarchies are still the underlying forces that bond heterogenous groups of people together. Leaders are then the key actors in identifying objectives, orienting followers, and providing a clear identity to organizations, by means of a shared vision (Gerbaudo, 2017; Bakardjieva et al., 2018).

### The Micro Level of Analysis: Main Categories

The studies that adopt a micro-perspective to the topic of leadership and digital technology can be classified in three different categories, depending on whether they focus on: (1) The increased complexity of C-level roles; (2) The skills e-leaders need; and (3) The practices for leading virtual teams effectively.

#### The Evolution of C-Level Roles

The huge impact that digitalization has had in the competitive business environment, transforming markets, players, distribution channels, and relationships with customers, has made it necessary for organizations to adopt a high-level strategic view on digital transformation. New responsibilities on the selection of digital technologies that will drive an organization's ability to remain competitive in a highly digitized world, are given mainly to its CEO (Gerth and Peppard, 2016). CEOs in the Digital Age assume the additional role of digital change agents and digital enablers, implying that they should recognize the opportunities offered by new technologies, and also push for their implementation. As suggested by Avolio et al. (2000), e-leaders have a fundamental role in appropriating the right technology that is suitable to their organizations' needs, but also in transmitting a positive attitude to employees about their adopting of new technology. CEOs are required to instill a digital culture into the top management team, involving it in actively sustain a digital change inside the organization (Gerth and Peppard, 2016). For this matter, a greater interaction is needed between the CEO and the Chief Information Officer (CIO), who will increasingly become a key player in the digital strategy definition and implementation, rather stay confined to an “IT-is-a-mess-now-fix-it” flavor of a role (Gerth and Peppard, 2016; Bekkhus and Hallikainen, 2017). Bekkhus and Hallikainen (2017) acknowledge an increased ambidexterity in the role of CIOs and develop a toolbox related to their role as gatekeepers and contributors. In order to reach their goals successfully, CIOs need to have a clear picture of both the characteristics of the digital strategy and the organizational needs it is supposed to satisfy. They should also carefully evaluate the readiness of the organization in every step of the changing process in order to adopt the proper pace. To avoid IT project failures, CEOs need to facilitate the recognition of the CIO's role, as well as promote collaboration between the CIO and other top managers (Bygstad et al., 2017).

As described before, digital technologies are not only used to support internal processes, but are also a way to build relationships with different actors in the external environment. Social media platforms in particular, are de facto powerful tools that C-level executives use to build communications channels with their followers (Obschonka et al., 2017). In a study analyzing the rhetoric of CEOs in social media, Grafström and Falkman (2017) suggest that CEOs' willingness and ability to construct a continuative dialogue through digital channels is a powerful way not only to manage organizational crisis but also to sustain the reputation and the image of the organization, positioning the brand and communicating the organizational values. Thus, as Tsai and Men (2017) unveil, by properly using social media, CEOs, as organizational leaders and spokespersons, can build trust, satisfaction and advocacy among their followers. According to the authors, digital technologies, and social media in particular, support CEOs in becoming “Chief Engagement Officers [who develop] meaningful interpersonal interactions and relationships with today's media savvy publics” (Tsai and Men, 2017, p. 1859). Even if CEOs have always been considered the personification of the organization, the rising need for transparency and authenticity has led CEOs to embrace the task of visible, approachable and social leaders who actively contribute to the engagement of followers and costumers (Tsai and Men, 2017).

In sum, C-level managers are faced with higher complexity of roles, related not only to new responsibilities in the digital strategy development, but also in the engagement of stakeholders across the organization's boundaries.

#### Leaders' Skills in the Digital Era

Defining what skills characterize leaders in the digital era has become a matter of interest in the literature. Studies analyze what are the relevant skills e-leaders should display in order to be effective. In line with the debate on universal and contingency theories, scholars ask to what extent the skills leaders need in order to lead e-businesses differ from the ones needed in traditional organizations (Horner-Long and Schoenberg, 2002). Most studies are based on expert surveys that engage with digital experts, managers, CEOs and Managing Directors of e-businesses (Lynn Pulley and Sessa, 2001; Horner-Long and Schoenberg, 2002; Schwarzmüller et al., 2018; Sousa and Rocha, 2018). A few studies also integrate expert surveys with interviews to IT specialists (Sousa and Rocha, 2018) and C-level managers (Horner-Long and Schoenberg, 2002).

Scholars agree that the introduction of digital tools affects the design of work, and, particularly, how people work together (Barley, 2015; Schwarzmüller et al., 2018). For example, digitalization opens up new possibilities such as virtual teams and smart working, introduces new communication tools, increases speed and information access, influences power structures, and increases efficiency and standardization. In order to steer organizations and help them reap the benefits from such digital transformations, leaders may need to develop a variety of different skills. We present below the main skills leaders need in the digital transformation era that have been highlighted in the literature.

##### Communicating through digital media

Global connectivity and fast exchange of information have created a much more competitive and turbulent environment for e-businesses, which must deal with rapid and discontinuous changes in demand, competition and technology (Horner-Long and Schoenberg, 2002). Scholars agree that the need for speed, flexibility, and easier access to information has facilitated the adoption of flatter and more decentralized organizational structures (Horner-Long and Schoenberg, 2002). In the digital context, knowledge and information become more visible and easier to share, allowing followers to gain more autonomy (Schwarzmüller et al., 2018) and to make their voices heard at all levels of the organization (Lynn Pulley and Sessa, 2001). As information becomes more distributed within the organization, power tends to be decentralized. Digital transformation allows real-time involvement of followers in many decision processes, increasing their participation. Therefore, leaders are expected to adopt a more inclusive style of leading (Schwarzmüller et al., 2018), asking for and taking into account followers' ideas into everyday decision making, using a two-way communication and interaction. Scholars maintain that followers' higher autonomy and participation can lead to a higher sense of responsibility for the work they are accountable for. This in turn should reduce the need for control-seeking behaviors previously exerted by leaders (Horner-Long and Schoenberg, 2002; Schwarzmüller et al., 2018).

At the same time, inspiring and motivating employees have become pivotal skills for leaders to master (Horner-Long and Schoenberg, 2002), and seem to be required to an even greater extent in order to encourage the continuous involvement and active participation of followers. Indeed, the same digital tools that provide autonomy to followers, may also drive them toward greater isolation (Lynn Pulley and Sessa, 2001). According to Van Wart et al. (2017) and Roman et al. (2018), some of the most common problems generated by the digitalization of organizations are worker alienation, weak social bonding, and poor accountability. It is therefore extremely important that leaders support and help followers in dealing with the challenges of greater autonomy and increased job demands, by adopting coaching behaviors that promote their development, provide resources, and assist them in handling tasks (Schwarzmüller et al., 2018).

Similarly, the ability to create a positive organizational environment that fosters a strong sense of collaboration and unity among employees has become vital for leaders to have. Yet, e-leaders' reliance on traditional social skills, such as the abilities of active listening and understanding others' emotions and points of view, may not be enough to warrant success in creating such environments. Rather, they need to integrate these social skills with the ability to master a variety of virtual communication methods (Roman et al., 2018). According to Carte et al. (2006, p. 326), “while leadership in the more traditional face-to-face context may emerge using a variety of mechanisms, in the virtual context it likely relies largely on the communication effectiveness of the leader.”

Roman et al. (2018, p. 5) label this skill as e-communication, and define it as “the ability to communicate via ICTs in a manner that is clear and organized, avoids errors and miscommunication, and is not excessive or detrimental to performance.” The leader needs to set the appropriate tone for the communication, while organizing it and providing clear messages. Moreover, the leader needs to master different communication tools, as their communication effectiveness depends largely on the ability to choose the right communication tool. Roman et al. (2018) provide a set of major selection criteria, which includes richness of the tool, synchronicity, speed of feedback, ease of understanding by non-experts, and reprocessing capability (ability to use the communication artifact multiple times in different venues). This ability allows to adapt the communication to the receiver preferences (as it would otherwise happen in a face-to-face interaction), so as to provide a variety of cues that enhance social bonding (Shachaf and Hara, 2007; Stephens and Rains, 2011), convey the right message to the target audience, and better manage urgency and complexity.

##### High speed decision making

One way in which the introduction of technology has changed the organizational life has been the greater need for speed. Scholars agree that e-business leaders are forced to make decisions more rapidly (Lynn Pulley and Sessa, 2001; Horner-Long and Schoenberg, 2002). This seems to suggest that decisiveness, and problem-solving abilities keep being extremely relevant for e-leaders, and may become even more prominent in the future (Horner-Long and Schoenberg, 2002). According to Lynn Pulley and Sessa (2001), never-ending urgency can create situations in which leaders needs to make decisions without having all information or without having time to think and analyze the problem properly, which may lead to falling back onto habitual responses, instead of creating novel and innovative ideas. To help navigate such situations, leaders need to be able to tolerate ambiguity, while being creative at the same time (Horner-Long and Schoenberg, 2002; Schwarzmüller et al., 2018). If it is true that the digital world forces leaders to examine problems and provide innovative answers at a faster peace, the use of information technology also allows them to make more informed decisions. Information systems can provide enormous amounts of real-time data. For this reason, the ability to process high volumes of fast-paced incoming and outgoing data (e.g., Big data), in order to analyze it, prioritize and make sense of the relevant information for decision-making, has become and will be even more relevant in the future. Recent research points out that leaders will increasingly need to collaborate with IT managers, providing directions for data analysis and offering meaningful interpretations of results (Harris and Mehrotra, 2014; Vidgen et al., 2017).

##### Managing disruptive change

The fast-paced technological evolution places high demands on organizations' ability to deal with continuously changing conditions and players. Lynn Pulley and Sessa (2001) highlight the constant need for organizations to adapt, foresee opportunities, and sometimes improvise, in order to maintain their competitiveness in the market. Under increasing pressure to innovate, leaders need to undertake an active role in identifying the need for change, as well as handling, and initiating change within their teams and organizations (Schwarzmüller et al., 2018). Horner-Long and Schoenberg (2002) findings confirm that e-leaders tend to show more entrepreneurial and risk-taking characteristics than leaders in traditional contexts. However, continuous change should not disrupt the focus and mission of the organization. While promoting a flexible and innovative attitude in the organization, the leader needs to clarify a common direction. Lynn Pulley and Sessa (2001) identify the ability to inspire and share a common vision about the future of the organization as one of the challenges of e-leaders, who are frequently confronted with the need for change. While acknowledging the importance of this skill, Horner-Long and Schoenberg (2002) did not find it to characterize e-leaders any more than traditional leaders.

##### Managing connectivity

Scholars maintain that e-leaders also need to foster their networking abilities. Beyond the need to explore and create networks to lobby for resources and stakeholder support (Horner-Long and Schoenberg, 2002) developing social interactions seems to play a key role in favoring innovation. As innovation becomes a top priority, leaders need to understand how to take advantage of networking opportunities (Avolio et al., 2014). The hyper-connected environment, in which leaders operate, especially with the ubiquitous use of social media and other digital platforms, provides new networking opportunities due both to an easier access to larger groups of individuals, and the possibility to establish connections through more immediate communication. New technologies and especially the advent of social networks might have reinforced the perception that being persistently part of the network is compulsory. As reported in Horner-Long and Schoenberg (2002, p. 616) “in the new economy some leaders do nothing but network - there is no commercial need. It is simply networking for networking's sake.” Although it is a general requirement to be able to create and maintain social relationships with various stakeholders, effective leaders differ specifically in the ability to recognize those relationships that lead to tangible benefits (Horner-Long and Schoenberg, 2002).

##### The renaissance of technical skills

Lastly, scholars underscore the increased value of technical competencies. This represents a shift from the latest paradigm established over the past four decades, whereby leadership primarily requires emotional and social intelligence competencies that enable the leader to understand, motivate and manage his team effectively. Notwithstanding, leaders also need to understand and manage the use of various technologies. Indeed, IT knowledge and skills have become high on demand requirements to operate in a digitalized environment (Horner-Long and Schoenberg, 2002). Furthermore, the mastery of current technologies must be balanced with the ability to stay current on the newest technological developments (Roman et al., 2018). This emphasizes the need to adopt a life-long learning approach to developing one's digital skills.

##### Developing leadership skills in the digital era

To lead in the era of digital transformation requires individuals to be both people-oriented and technically minded (Diamante and London, 2002). These two skills often characterize very different profiles of people that, yet, need to come together in order to implement an effective digital transformation in their organization. The case study presented by Coutu (2000), highlights the need to establish a profitable exchange relationship between leaders of people-oriented (e.g., sales), and IT functions, in order to create a cross-functional and cross-skill contamination. Systematic knowledge dissemination from the individual to the group is highlighted as the most effective way to spread knowledge and expertise across the organization (Boe and Torgersen, 2018). Coutu (2000) addresses how this cross-skill contamination can be performed, by means of implementing reverse-mentoring programs. Nonetheless, the author uncovers the problem of potential generational conflicts, whereby newer generations, who tend to be more knowledgeable and skilled in digital technologies, may gain informational power over others, generating concern and skepticism in older, change averse, individuals (Coutu, 2000).

Studying modern military operational environments, Boe and Torgersen (2018) highlight the need to lead under volatile, uncertain and complex situations, characteristics they find similarly describe the context of modern e-businesses. According to the authors, leadership training needs to combine both technology and change, creating simulations of scenarios in which ambiguous information and improvisation create complex and uncertain conditions.

One way in which exposure to technology and simulations can be combined is through training in virtual spaces (Lisk et al., 2012; Lu et al., 2014). In large community games, leaders may have to recruit, motivate, reward, and retain talented team members. They have to make quick decisions that may affect their outcomes in the long-run, for which they need to analyze the environment in order to build and keep their competitive advantage (Avolio et al., 2014). Lu et al. (2014) adopt experiential learning theory (Kolb and Kolb, 2005) to explain e-leadership skills development, referring to activities in which learning is performed in a virtual context. Their study attempts to empirically examine the transferability of virtual experiences into in-role job situations. Results show partial association between virtual games behaviors and hierarchical position of the participants, however, conclusions concerning the transferability of certain skills or experiences gained in virtual games may be highly affected by reverse causality. Ducheneaut and Moore (2005), conduct a virtual ethnography to show that people participating in multiplayer role-playing games train behaviors related to networking, management and coordination in small groups. However, in a recent review on the use of games, based on digital tools or virtual realities, for training leadership skills, Lopes et al. (2013) highlight a general lack of theoretical grounding in the development and analysis of virtual games. Moreover, they find extant studies rarely show these games affect leadership skill outcomes (Lopes et al., 2013). Robin et al. (2011) find that while simulations facilitate learning, they do not seem to lead to better results than traditional methods. The authors suggest simulations' main advantage lies in the possibility to enable learning in situations where it would otherwise be difficult or impossible. They thus propose the use of a combination of traditional and technology-based training to achieve the most effective learning outcomes.

#### Leading Virtual Teams

The introduction of digital tools has enable the organizational structure to become not only flatter and decentralized, but also dispersed. One way in which digital technology has shaped organizational life and people management has been by enabling the potential use of virtual teams. Virtual teams are defined as “interdependent groups of individuals that work across time, space, and organizational boundaries with communication links that are heavily dependent upon advanced information technologies” (Hambley et al., 2007, p. 1). They have become increasingly pervasive in the last years, especially in multinational organizations (Gupta and Pathak, 2018).

Indeed, several benefits of virtual teams have been acknowledged in the literature. First, the use of virtual teams has allowed for a dramatic reduction of travel times and costs (Bartol and Liu, 2002; Bergiel et al., 2008). Second, it has enabled teams to draw upon a varied array of expertise, regardless of location (Jawadi et al., 2013), making it easier to access and recruit talent across the globe. Third, by facilitating the heterogeneity of team members, it has fostered creativity and innovation, due to the possibility of combining different perspectives (Gupta and Pathak, 2018).

Despite its advantages, certain specificities of virtual teams' challenge the traditional way in which teams are managed and led. For instance, virtual teams are characterized by geographical and/or organizational distance. This implies that leaders cannot physically observe team members' behavior nor rely on verbal cues, facial expressions, and other non-verbal communication in order to understand the team's thoughts, feelings, moods and actions. This is considered one of the biggest barriers to developing and managing interpersonal relationships (Jawadi et al., 2013). The heavy dependence on ICT may lead to communication problems, such as failing to distribute information to all team members, understand or convey the level of urgency or importance of the information, and interpret silence (Cascio and Montealegre, 2016). Geographical dispersion often implies cultural diversity between team members, which may affect leaders' ability to build and maintain team spirit and trust (Gupta and Pathak, 2018). According to Sullivan et al. (2015), space may suppress leadership capacity, even in situations of shared leadership. Moreover, virtual teams are subject to time differences.

In order to overcome these challenges, virtual team leaders need to adopt specific behaviors and practices. One of the most important practices highlighted in the literature involves the setting and periodical revision of communication norms within the team (Jawadi et al., 2013). Instead of focusing on behavioral norms, as in traditional teams, virtual teams require a clear definition of the norms pertaining to their use of communication tools, through witch information flows and activities are performed. Clear communication norms entail a number of advantages for virtual teams, such as: correct exchange of information, regular interaction and feedback, less ambiguity about teamwork processes, better monitoring of each member's contributions, faster detection of problems and mistakes. Moreover, because leaders play a fundamental role in enabling and mediating the communication between team members, they are able to lead them in the construction of a common language. This involves gaining a deep understanding of the underlying meaning of words and expressions used in the team. The mutual understanding of the organizational and social context in which each team member is embedded facilitates this process (Plowman et al., 2007; Bjørn and Ngwenyama, 2009; Rafaeli et al., 2009).

As mentioned in the previous section, virtual team leaders also need to be able to choose the right communication tools and navigate well through their functionalities and the interactivity across various tools, if they are to avoid disruptions in communication and achieve a more vivid and open communication that favors positive team member relationships (Jawadi et al., 2013). While synchronous communication is considered more appropriate to manage complex, interdependent tasks (Hambley et al., 2007), asynchronous instruments may allow for team members with different backgrounds to adopt their own pace in processing others' ideas or generating new ones (Malhotra et al., 2007). Moreover, asynchronous communication facilitates a continuous flow of information and the ability to work for a greater number of hours (Gupta and Pathak, 2018). Furthermore, leaders need to use multiple channels with different levels of richness (Hambley et al., 2007). According to Hambley et al. (2007), “a rich medium allows for transmitting multiple verbal and nonverbal clues, using natural language, providing immediate feedback, and conveying personal feelings and emotions.” A richer tool is supposed to lead to better team cohesion. Yet, the authors found mixed results in terms of the association between constructive interaction and task performance (Hambley et al., 2007).

Virtual teams often group together individuals from different educational, functional, geographical and cultural backgrounds. On one hand, such heterogeneity should promote innovative solutions, but on the other hand, it may also undermine collaboration. A virtual team leader thus needs to have good cross-cultural skills (Schwarzmüller et al., 2018), to identify different cultures' characteristics and understand similarities and differences across cultures. Especially at the early stages of a virtual team's lifecycle, the leader needs to assure that the diversity of team members is understood, appreciated, and leveraged. As virtual teams do not usually have the chance to enjoy in-person informal activities typically used to share personal characteristics and abilities and foster team building, the leader needs to share and manage personal information virtually and ensure the team has a clear understanding of each team member's expertise and skills (Malhotra et al., 2007). Once the diversification of skills is acknowledged, virtual teams can also benefit from a clear distribution of roles and tasks (Jawadi et al., 2013). Especially if virtual teams adopt asynchronous communication tools, tasks and schedules need to be clearly defined to avoid delays due to task misallocation or overlapping.

According to Malhotra et al. (2007), virtual teams may also engage in practices aimed at digitally monitoring the team activity, relying on remote monitoring of virtual communication and participation, as well as document posting. However, Jawadi et al. (2013) notice how monitoring and controlling mechanisms may be negatively perceived by team members. Indeed, their findings show that behaviors directed at monitoring and coordinating team interactions are not associated with higher leader-member relationship quality. According to Carte et al. (2006), high performing virtual teams are characterized by monitoring behaviors, but only when these are shared between members. Although, traditional performance appraisal and monitoring mechanisms are being replaced by alternative systems that rely on real-time digital feedback, the key features that characterize effective face-to-face feedback have been kept (Petrucci and Rivera, 2018).

Perhaps the best measure of impact of the pervasive adoption of virtual teams in organizations has been the extensive accumulation of literature focused on studying the phenomenon, alongside its antecedents, challenges and outcomes. As our study reveals, scholars have identified a number of best practices, whereby virtual team leaders become the key players in charge of resolving the challenges posed by physical and organizational distance.

However, especially when considering virtual teams, there has been a shift in the literature to steer away from traditional notions of leadership as being assigned to one individual, toward focusing on new conceptualizations of shared and distributed leadership. Virtual teams, which are often cross-functional, are indeed characterized by a relative absence of formal hierarchical authority (Pearce et al., 2009). In the same way that the need for speed in responding to accelerated environmental change and higher connectivity led to the development of virtual teams, that same need may be driving the flattening of hierarchical structures toward more evenly distributed, shared and empowered leadership among virtual team members (Pearce et al., 2009). As such, virtual teams are often left alone to shape and define their own leadership style, which may encourage all team members to perceive themselves as leaders and drive the collective development of leadership skills (Gupta and Pathak, 2018). In these so called self-managing work teams (SMWTs; Manz and Sims, 1987; Druskat and Wheeler, 2003), decisions and leadership responsibilities are equitably allocated among team members, who are also engaged in supporting and accompanying each other in the accomplishment of their tasks. The concept of shared leadership does not necessarily imply the rejection of a “formal” leader, but introduces the idea that any team member may be a leader, and as such, is expected to assess the team in its context and assert what is best for the team: whether to volunteer himself as team leader or empower any fellow team member(s) to serve the team as leader(s). This process leads to the creation of a shared understanding of both the leadership responsibilities and the power dynamics within the team (Grisoni and Beeby, 2007; Hoch and Kozlowski, 2014; Hoegl and Muethel, 2016).

## Toward The Future: Research Directions

Despite the urgency felt by scholars to understand how leaders keep the pace with technological change, the literature seems to lack a shared approach in studying and theorizing about this phenomenon. Although researchers have been introducing relevant new concepts, such as e-leader and e-organizations, there is a shortage of well-established and consensual definitions in the literature. Our review reveals scholars have relied on several leadership theories to explain the relationship between leadership and digital transformation. However, we question whether theories based on traditional views of industrial organization and business, that still prevail in the literature, are the most suitable to comprehend the multifaceted phenomenon of digital transformation and its impact on all matters leadership of organizations, communities, teams, and even self. As suggested by Kahai et al. (2013), scholars may need to go beyond traditional leadership theories to explain the impact digitalization exerts on leadership and leaders. Are the existing theories in social sciences able to explain the antecedents, characteristics and outcomes of this disruptive phenomenon or do we need new theoretical lenses to make sense of how leaders may respond to this change?

One of the most complex and pressing issues concerns e-leaders (un)ethical behaviors. Notably, the higher risk leaders now face of engaging in unethical uses of personal and sensitive information, or the inexistence of a code of conduct for ethical leadership behavior are critical concerns to raise in any debate of e-leadership (Lee, 2009). Collaboration through digital technologies brings about new questions regarding the role leaders may play in the digital environment. What is the role of leaders in guiding an ethical appropriation of digital technologies? What can e-leaders do in order to be an example and instill an ethical culture within their followers? How do digital tools such as social media and online communities and forums change the conditions under which interactions occur and how do these affect the maintenance of ethical behaviors? These are questions that future research is pressed to answer. While the theoretical debate has already started to address some of these questions, empirical research remains considerably underdeveloped.

The present review uncovers a shortage of contributions addressing the role that institutions play in supporting ethical behaviors of leaders. In particular, what remains unclear is whether and how leaders will be prepared to face the new wave of data and policies that affect their ability to manage privacy and regulatory issues. Studies in this area are thus highly encouraged.

The leader-follower relationships mediated by ICTs can also be affected by concerns for privacy and information that the parties do not want to share. Social media interactions, for example, leave digital footprints that can be monitored by leaders and organizations, which may compromise the interactions and responses of followers that feel their privacy is at risk. The same can be said regarding the instruments that digital technologies provide for tracing personal productivity. Project management applications, for instance, trace individual contributions to a certain project, but can challenge an impartial evaluation if the relationship between individual effort and contribution to the results is not clear, thus putting into question the trust in the relationship with leaders. Future research should consider these aspects and work toward a broader comprehension of how to balance the need for higher transparency in ICT- mediated relationships with followers' higher autonomy and need for privacy.

We acknowledge that the introduction and use of digital tools it strictly linked to organizational cultures that value the use of technology and establishes the readiness of organizations to successfully implement digital tools. Therefore, we suggest further research needs to investigate the extent to which culture affects the selection and effective implementation of digital technologies within organizations. Answering to this question also provides relevant information on how digital technology alters organizational identity and shapes new organizational boundaries. Exploring this line of inquiry using both theoretical and empirical approaches, may inform the creation of new organizational identities, and their relationship with different types of organizations and institutions.

Since digitalization is enabling a growing propensity to share information, organizational boundaries are becoming more fluid and expanding outside the formal organization. Hence, collective forms of leadership are expected to increase. Notably, distributed or shared leadership is supposed to gain momentum, especially if it is considered a better fit to the characteristics of virtual teams, such as the informal nature of its communication channels, task interdependence and team member autonomy (Avolio et al., 2014; Hoch and Kozlowski, 2014). What remains unclear is the role that leaders play in recognizing and encouraging distributed leadership in teams. Moreover, how much does the success of shared leadership styles depend on the organizational culture? What is the effect of shared leadership on virtual team dynamics? We claim that these are questions that should be explored with greater detail in the future.

Networked organizations, as well as the rise of virtual teams, speak volumes about the endless connectivity possibilities that digital technology has enabled. However, empirical studies on virtual teams also highlight that digital tools and media can disconnect individuals and undermine established power dynamics. Despite the relevance of increased connectivity, only a few studies adopt a network approach to understand how leaders and followers are interconnected to one another.

Literature has already acknowledged that the lack of face-to-face interactions makes the task of leading virtual teams a more complex job (Purvanova and Bono, 2009). Indeed, the physical and cultural distance that characterizes virtual teams threatens the ability to build trust, create commitment and enhance cohesion among team members (Hoch and Kozlowski, 2014. As suggested by Lee (2009) trust in virtual teams is related to ethics: the way in which leaders and team members behave, the extent to which they demonstrate transparency when interacting with others, the integrity and compliance to the rules and procedures of the organization and the team are key issues that should not be neglected. However, little is known about the methods and behaviors that effective leaders can adopt in order to build trust in virtual teams. Literature on this topic needs contributions that focus specifically on the process of trust creation in virtual teams, describing its characteristics and mechanisms and informing about which digital tools can be used to support such process. Indeed, along with the ability of creating trust among team members, virtual team leaders are required to have the ability of choosing and exploiting the right communication tools (Jawadi et al., 2013; Roman et al., 2018). Future research should try to uncover the effect different characteristics of communication tools may have on team dynamics and leader-followers relationships.

The lack of face-to-face interaction also creates new challenges in the deployment of social skills. Processes related to interpersonal understanding may be inhibited by distance and by the use of interfaces. Indeed, comparing traditional face-to-face teams and pure virtual teams, Balthazard et al. (2009) found that leader characteristics that are easier to perceive from nonverbal cues, such as personality traits, predicted the emergence of transformational leadership in face-to-face teams, but not in computer-mediated teams. Considering the importance of social understanding and affect-based perceptions, we encourage future research that analyzes the ways in which leaders can create positive emotional contagion, through technology. For example, it could be interesting to inquire whether the use of facial/emotional recognition devices (Pentland and Choudhury, 2000), and affective haptics (Arafsha et al., 2012) can contribute to interpersonal emotional understanding and sharing, and how it affects leader-follower relationships and team dynamics. Balthazard et al. (2009) found written communication quality to be positively related to the emergence of transformational leadership in virtual teams. Indeed, the increasing adoption of written communication-based tools such as chats, social media, or document sharing platforms, calls for the use of linguistic analysis of online communication to understand how leaders effectively instill emotions, convey their vision, or communicate urgency through text.

As suggested by Avolio et al. (2014), leadership in the digital world may be influenced by gender. Men and women may adopt different criteria in choosing which technologies to adopt. However, this topic of research has earned little attention in the literature. We claim that other studies are needed to investigate more in depth gender differences, and its effect on organizational outcomes.

Another topic that future researcher needs to address regards the way in which leaders can develop the skills needed to perform in the digital era. Some scholars maintain virtual games might be useful instruments to foster both social and technical skills (Ducheneaut and Moore, 2005; Lu et al., 2014). However, findings have not yet showed whether virtual games have a clear effect on social and digital skills development. We suggest future research could inspect what types of virtual behaviors foster team engagement and higher team performance in multiplayer virtual games, while examining the role of these variables in organizational settings. Other scholars propose digital natives and technical experts in organizations may be engaged in the training of those who are less familiar with or demonstrate a negative attitude toward the adoption of technology, for example by means of reverse mentoring programs (Coutu, 2000). However, conditions that can favor a successful digital transformation of organizations should be analyzed. The technological skill advantage of young generations may destabilize traditional power relations. A closer look to this phenomenon is suggested.

In a digital world where physical presence is becoming unnecessary, the possibility that some leadership responsibilities begin to be performed by AI-based technology is not unrealistic. A tough debate is raising awareness as to whether robots can be programmed to express emotions and how this fosters the possibility that robots may be better leaders than humans (Avolio et al., 2014). Complementing the literature that has so far stressed the importance of emotions and emotional intelligence for leaders' performance (see for instance Boyatzis, 2006; Boyatzis et al., 2017), future research should shed light on whether and how robots, algorithms and technological tools substitute or complement leaders.

Even if macro and micro level of analysis are explored by social science scholars, management literature would still lack the analysis of the phenomenon of leadership and digitalization at the meso-level. A promising way of combining micro and macro levels of theorizing might be to introduce a multiple level of analysis. Some of the papers in our dataset move toward this direction, however, it is not clear how digitalization is affecting relationships between diverse organizations.

Finally, from a methodological point of view, our study shows a plethora of methods employed by scholars to analyze leaders' behavior (Hambley et al., 2007; Malhotra et al., 2007; Jawadi et al., 2013), leaders' skills (Horner-Long and Schoenberg, 2002; Roman et al., 2018), or technology adoption (Bartol and Liu, 2002; Weiner et al., 2015). If on one hand, this richness provides a portfolio of techniques that scholars could use depending on the subject of analysis, on the other hand, it confirms that there is still a confusion about how to monitor this recent phenomenon. Moreover, we observe that contributions are confined within their own disciplinary frontiers. For instance, social movements literature, that mainly draws on qualitative methods such as ethnography, case study, and interviews, should inform organizational scholars how to observe power relations within companies. Extant contributions investigating what are the skills leaders facing the digital transformation require are based mainly on experts' surveys and interviews. Literature reveals a lack of empirical research which examines the relationship between identified leadership skills and successful performance in highly digitalized organizations. Future studies should also take into account how much this relationship may be affected by the context in which the leader operates.

## Conclusion

Nowadays, digital transformation is an unavoidable choice for any company, regardless of size or sector. Leaders cope with new tools on a daily basis and they make decisions according to the data they have access to. Therefore, we highly encourage future research to shed more light on the effect of digital transformation on leadership, both at organizational and individual level. If the debate about the relationship between human beings and machine is not a recent one (Turing, 1950), not to management literature, nor social sciences in general, the relationship between digital transformation and leadership requires updated lenses. This systematic review offers a structured framework of a promising field, and we hope it will help future research generate coherent efforts to garner novel and relevant knowledge in this research topic.

The purpose of this review was 3-fold. First, we discussed how leadership in the digital era has been conceptualized, reviewing the theoretical perspectives that have been used in prior research. Our review did not reveal a strong unifying theory of the relationship between leadership and digital transformation, thus calling for more attention to theoretical contributions.

Second, we mapped the academic debate on the relationship between digital transformation and leadership, organizing and structuring the main emerging themes at macro and micro level of analysis. We observed that both contributions with micro and macro approaches underscore that information technology and strategic management need greater alignment. Digital transformation is successful in the long term when the overall organizational objectives match the need to adopt a new digital tools or instruments. In a similar vein, individuals embrace technological advancement only when they perceive it is relevant to their tasks. It is an important responsibility of the leader, particularly of C-level leaders, to steer this strategic alignment and the proliferation of a digital culture.

In a networked economy, the digital transformation has led organizations to open their boundaries, and connect with other industries, stakeholders, and customers, to generate innovation. From a micro perspective, this openness is also required by leaders who need to invest in networking. This means to be “out there” (Grafström and Falkman, 2017), present in the network (Gordon, 2007), and willing to communicate with different types of stakeholders, through digital tools and social media. Especially for leaders, the digital tools are no longer a distant container of everyday life; rather, they are instruments in which everyday life emerges (Gordon, 2007).

Although the introduction of digital tools influenced organizational boundaries and leadership boundaries, for instance favoring the development of concepts such as shared leadership, studies show that trust among members and employees is still achieved and maintained through leaders' intervention (Carte et al., 2006). Cascio and Montealegre (2016, p. 356), reminds us that inspirational leaders will remain pivotal in making the right decisions, as “humans will continue to enjoy a strong comparative advantage over machines.” However, the growing development and use of AI-based technology to make decisions, calls for a closer understanding of what leadership will mean in the future. Growing ethical concerns related to the application of AI in managerial activities as well as to the appropriation of technology and data are becoming an urgent topic to address.

To overcome the challenges derived from the digital transformation, leaders are required to develop a combination of digital and human skills, mainly related to the ability to communicate effectively in a digitalized context, create cohesion between geographically distant followers, foster initiative and change attitudes, and deal with complex and fast problem solving.

Third, we highlighted the current gaps and open questions in the literature, and laid out a future research agenda that targets opportunities for the empirical and theoretical advancement of knowledge.

While our review is timely and includes the most recent contributions, some limitations should be considered and overcome in future studies. First, since our concern was to map prior research, we have not provided detailed propositions to the suggested categories, a void that should be addressed by future studies. The second concern regards the sample. We drew from the Scopus database only. Albeit we checked other databases to avoid potential bias, we may have missed some relevant articles contained elsewhere. Third, despite the rigorous procedure of our systematic review, a limitation is ascribed to the inclusion of only peer-reviewed articles and conference proceedings. A future review should also include industry research reports, professional outlets publishing research-based findings, and other non-pear reviewed manuscripts to better clarify how the multidimensional phenomenon of digitalization is affecting organizations and leadership. Finally, we excluded, as per our boundary conditions, articles that considered organizations as leaders in the digital transformation, and studies that discussed about digital platforms. Future studies should adopt a broader overview of the macro-organizational and strategic effects in order to understand how digital transformation is implemented across different organizations, communities and teams.

## Author Contributions

LC and EB contributed conception and design of the study. LC, EB, and RZ organized and analyzed the database. LC and EB wrote the first draft of the manuscript. RZ wrote sections of the manuscript. All authors contributed to manuscript revision, read and approved the submitted version.

### Conflict of Interest Statement

The authors declare that the research was conducted in the absence of any commercial or financial relationships that could be construed as a potential conflict of interest.
